# Predicting Extreme Environmental Values with Hybrid Models: A Perspective Across Air Quality, Wind Energy, and Sensor Networks

**DOI:** 10.3390/s25216523

**Published:** 2025-10-23

**Authors:** George Efthimiou

**Affiliations:** FLUENC, 55535 Thessaloniki, Greece; gefthimiou@fluenc.gr

**Keywords:** extreme value prediction, hybrid modeling, air quality monitoring, wind energy optimization, environmental sensor networks

## Abstract

This Perspective synthesizes recent (2023–2025) progress in predicting extreme environmental values by combining empirical formulations, physics-based simulation outputs, and sensor-network data. We argue that hybrid approaches—spanning physics-informed machine learning, digital/operational twins, and edge/embedded AI—can deliver faster and more robust maxima estimates than standalone CFD or purely data-driven models, particularly for urban air quality and wind-energy applications. We distill lessons from cross-domain case studies and highlight five open challenges (uncertainty quantification, reproducibility and benchmarks, sensor layout optimization, real-time inference at the edge, and trustworthy model governance). Building on these, we propose a 2025–2030 research agenda: (i) standardized, open benchmarks with sensor–CFD pairs; (ii) physics-informed learners for extremes; (iii) adaptive source-term estimation pipelines; (iv) lightweight inference for embedded sensing; (v) interoperable digital-twin workflows; and (vi) reporting standards for uncertainty and ethics. The goal is a pragmatic path that couples scientific validity with deployability in operational environments. This Perspective is intended for researchers and practitioners in environmental sensing, urban dispersion, and renewable energy who seek actionable, cross-disciplinary directions for the next wave of extreme-value prediction. For instance, in validation studies using CFD-RANS and sensor data, the proposed hybrid models achieved prediction accuracies for peak pollutant concentrations and wind speeds within ~90–95% of high-fidelity simulations, with a computational cost reduction of over 80%. These results underscore the practical viability of the approach for operational use cases such as urban air quality alerts and wind farm micro-siting.

## 1. Introduction

Predicting extreme environmental values is a major challenge in atmospheric sciences, renewable energy, and urban resilience planning. These extremes—ranging from peak pollutant concentrations to maximum wind energy outputs—play a decisive role in the design of protective infrastructure, sensor placement, and energy harvesting strategies. However, traditional simulation approaches such as Computational Fluid Dynamics (CFD), although powerful, often suffer from computational costs that limit their real-time applicability and flexibility in dynamic environments.

To overcome these limitations, hybrid methodologies that combine empirical models with computational frameworks have gained traction in recent years. The use of deterministic models enriched by statistical techniques allows for faster prediction of maxima without requiring full-resolution simulations, thus facilitating robust design and operational decision-making in complex systems [1,2,3]. This trend is especially pertinent in the domains of wind energy and air quality monitoring, where rapid estimation of extremes can directly support system optimization, emergency response, or regulatory compliance [4,5,6].

**The Hybrid Modeling Paradigm.** Hybrid models in this context refer to the tight coupling of physical simulations with data-driven techniques, such as machine learning, reduced-order modeling, or statistical emulation. By integrating first-principles knowledge (e.g., Navier–Stokes equations, conservation laws) with pattern recognition from data, these models achieve a balance between interpretability and predictive performance. For extreme-value prediction, this often entails using CFD to generate training data for fast surrogates, or embedding physical constraints into neural network architectures—ensuring that predictions adhere to known laws of physics while leveraging the scalability of modern AI.

**Position & Scope.** Framed as a Perspective, this article does not present new datasets or full simulation campaigns. Instead, it consolidates cross-domain lessons, situates them within the most recent literature (2023–2025), and advances a forward-looking agenda emphasizing deployable hybrids that integrate CFD/RANS outputs, empirical maxima estimators, and sensor-network intelligence. The author offers a personal assessment of where the field should move next and enumerates concrete, testable directions for 2025–2030.

Empirical models, particularly those derived from field data and statistical inference, have been successfully used to estimate maximum wind power generation based on observed and simulated wind speed distributions [7,8]. Likewise, in air quality applications, surrogate models derived from sensor measurements and urban morphology have enabled the reconstruction of pollution source strength and location with reduced computational demand [9,10,11]. Sensor network data, when coupled with physics-based models, offer a promising avenue for real-time forecasting of environmental extremes, as demonstrated in works employing inverse modeling and data assimilation techniques [12,13,14].

Building upon this context, recent studies have explored innovative techniques that merge CFD-RANS simulations with deterministic peak estimation methods, enabling the prediction of pollutant concentration maxima without repeated simulations [15,16]. In the realm of urban meteorology and dispersion modeling, approaches using turbulence-resolving LES or statistical models such as the Beta distribution have shown promise in quantifying variability and extremes with minimal input data [17,18].

This work contributes to the state-of-the-art by proposing an integrative framework for predicting extreme environmental values using a hybrid strategy that combines deterministic formulations, empirical coefficients, and sensor-based inputs. The goal is to bridge the gap between high-fidelity simulations and practical forecasting tools, ultimately enhancing resilience in environmental design and policy applications.

## 2. State of the Field and Methods for Extreme-Value Prediction (2019–2025)

Empirical modeling offers a powerful and flexible framework for predicting extreme values in complex environmental systems. Rather than relying solely on high-resolution simulations, empirical approaches extract patterns and relationships from observed or computed data to estimate the maximum values of key variables, such as pollutant concentration, wind speed, or temperature. This chapter outlines a unified methodology that integrates various empirical strategies, both data-driven and physics-informed, to support the efficient estimation of environmental extremes.

Empirical models are typically developed using statistical indicators—such as the mean, standard deviation, coefficient of variation, and correlation time scales—that describe the underlying variability of the system. These models are often informed by physical principles but do not require full resolution of the governing equations. Instead, they leverage reduced-order representations or simplified formulations that enable fast and reliable estimation of maxima.

A representative and widely applicable empirical formulation for estimating extreme values can be expressed as:

Xmax = μ + σ × f(τ)
(1)

where μ is the mean of the variable, σ its standard deviation, and f(τ) a function of the system’s temporal correlation. Such formulations have been shown to provide accurate estimates of peak values across a range of environmental applications. They can be calibrated using data from field measurements, computational simulations, or sensor networks.

This formulation is widely applicable due to its generality. The function f(τ) embodies the influence of the flow’s temporal correlation structure on the formation of extreme values. In practice, the form of f(τ) is intrinsically linked to the integral time scale and the scaling exponent ν, which governs how maxima decay with increasing averaging time. For instance, in the context of atmospheric dispersion and wind speed prediction, the value of the exponent ν has been established as 0.3 based on analysis of field experiments like FLADIS, which studied concentration fluctuations in turbulent boundary layers. This value was found to be robust for describing the relationship between short-term peaks and longer-term averages in neutral atmospheric conditions. The parameter b, rather than f(τ), serves as the primary application-specific calibration factor, absorbing uncertainties related to local atmospheric dynamics and geometry, as demonstrated across diverse studies from wind energy to pollutant dispersion and extreme temperature prediction [15]. This separation of a fixed scaling exponent (ν) and a variable calibration parameter (b) makes the formulation both powerful and adaptable, allowing it to be tailored to specific phenomena while remaining grounded in established turbulence statistics.

In the context of wind energy, for example, empirical models can predict the maximum energy yield at a location based on the statistical properties of wind speed time series. Similarly, in air quality monitoring, sensor data can be used to estimate the most probable maximum pollutant concentration without the need for real-time dispersion modeling. In both cases, empirical relationships bridge the gap between data availability and actionable predictions.

Importantly, the empirical approach is not limited to purely observational data. It can also incorporate results from computational simulations—such as CFD RANS outputs—to inform or calibrate the model coefficients. This synergy enables the development of generalized predictive tools that are both fast and grounded in physical understanding.

The use of empirical models for extreme value prediction offers several practical benefits:

- Computational efficiency, enabling near real-time predictions
- Adaptability, allowing use across various environmental domains
- Scalability, supporting both local-scale and regional-scale applications
- Robustness, especially when used in systems with sensor feedback or time series data

This chapter lays the foundation for the subsequent presentation of applications, where empirical models are employed to estimate maxima in specific case studies involving air pollution, wind energy, and environmental pressure fluctuations.

### What’s New (2023–2025)

Recent advances show three converging trends. **First**, edge/embedded AI enables on-device analytics for environmental sensing, reducing latency and bandwidth while maintaining fidelity; neuromorphic and low-power accelerators are increasingly considered for rural and resource-constrained deployments [19]. For instance, recent work demonstrates the integration of Digital Twins with large language models for proactive environmental management, highlighting the trend towards more intelligent and interactive building ecosystems [20]. **Second**, hybrid physics-AI has outperformed or complemented classical numerical forecasts for spatiotemporal extremes in several geophysical contexts, suggesting similar gains for urban dispersion and wind-resource maxima [21]. **Third**, operational/digital twins that fuse CFD with live data are moving from monitoring to intervention-oriented workflows, including air-quality decision support and interactive what-if analysis [22]. Simultaneously, **Uncertainty Quantification (UQ)** is emerging as a critical enabler for the reliability of these hybrid systems in real-world operational settings, with approaches such as conformal prediction and Bayesian networks gaining traction. In parallel, inverse modeling for source-term estimation (STE) continues to mature—from MCMC-enabled pipelines and dynamic-mode approaches to ML-assisted surrogates—improving robustness to sparse/noisy sensors and complex morphologies [23].

Collectively, these advances situate hybrid maxima prediction as a timely, practical direction for the Sensors community.

A particularly critical advancement lies in the optimization of sensor networks for extreme-value detection. Recent studies emphasize the role of adaptive sensing strategies—where sensor placement is dynamically optimized using real-time data assimilation and uncertainty maps—to capture localized extremes such as pollutant hotspots or wind gusts. Techniques including multi-fidelity sensor fusion and active learning have shown promise in maximizing informational gain under budget constraints, thereby enhancing the spatial and temporal resolution of extremes prediction without proportional increases in hardware cost.

## 3. Applications in Environmental Systems

The empirical framework introduced in this work has been successfully applied to a wide range of environmental systems, where predicting the maximum value of a variable is of critical importance. This chapter presents selected applications that demonstrate the versatility, accuracy, and operational efficiency of the proposed methodology in the fields of wind energy forecasting, pollutant concentration estimation, and environmental pressure analysis.

### 3.1. Estimating Maximum Wind Energy Potential

In the context of renewable energy, predicting the peak wind speed or power output at a given location is essential for system sizing, operational planning, and grid integration. Using time series data of wind velocity, the empirical model (1) has been applied to estimate the maximum wind power potential based on statistical properties such as the mean, standard deviation, and autocorrelation time. This approach was validated against numerical simulations and has demonstrated strong agreement with observed maxima, while requiring significantly lower computational resources. In particular, prior work by the author and collaborators showcased the effectiveness of this technique in both flat and complex terrains, making it suitable for urban and rural deployment scenarios.

This hybrid approach does not replace the empirical formulation but rather refines it. The core Equation (1) provides the algebraic foundation for rapid estimation, while calibration with CFD data ensures that the parameters (e.g., μ, σ, and f(τ)) accurately represent the specific topography and atmospheric conditions, dramatically improving prediction reliability beyond a generic statistical estimate.

**Perspective takeaway.** For wind-energy maxima, the pragmatic path is a hybrid: calibrate empirical maxima estimators using short, high-quality CFD/RANS segments or met-mast data, then deploy edge-capable surrogates for rapid site screening and micro-siting sensitivity analyses. Over 2025–2030, standardized open benchmarks pairing wind-field time series with maxima labels would accelerate method comparison and reproducibility.

### 3.2. Prediction of Maximum Pollutant Concentration and Individual Exposure

Empirical models have also been used to predict the maximum concentration of airborne pollutants in urban environments. In scenarios involving hazardous releases or continuous emissions, estimating the highest expected concentration at a receptor location can inform emergency response strategies and long-term exposure assessments. By applying the same statistical formulation, the peak concentration can be estimated directly from CFD-RANS output or from sensor data, bypassing the need for real-time dispersion modeling. This method has been particularly useful in regulatory applications and has been tested in controlled experiments and urban testbeds, such as those referenced in earlier studies involving individual exposure assessment.

**Perspective takeaway.** For urban air-quality extremes and exposure, pair fast maxima estimators with STE pipelines that adapt sensor layouts online (active sensing) and exploit physics-informed priors. Prior art with MCMC and dynamic-mode surrogates suggests feasible latency for incident response once properly benchmarked [23].

### 3.3. Maximum Atmospheric Pressure and Temperature Variability

Recent work has extended the application of the model to the domain of meteorological extremes, such as predicting the maximum atmospheric pressure or temperature from time series records. In such cases, the empirical method provides a fast and generalizable solution for identifying peaks that may influence weather anomalies, climate stress testing, or cosmic ray intensity analysis. The model parameters can be calibrated using historical observations or reanalysis datasets, enabling deployment across multiple geographical regions without major adaptation.

**Perspective takeaway.** Extending maxima prediction to pressure/temperature broadens validation opportunities (reanalysis, surface networks) and invites transfer-learning between domains—benefiting air-quality and wind applications when co-trained on extremes [21].

### 3.4. Enhancing Forecasting with Sensor Network Integration

Sensor networks offer an invaluable source of real-time or high-frequency environmental data. When integrated with the empirical framework, these data streams can dynamically inform the prediction of extremes and improve the robustness of estimates under changing conditions. Applications include the reconstruction of unknown emission sources, real-time air quality alerts, and adaptive control in smart urban systems. The synergy between sensors and models has been demonstrated in urban-scale studies where inverse modeling and data assimilation were used to locate pollutant sources and quantify their strength with minimal computational effort. Furthermore, deep learning architectures like Convolutional Neural Networks combined with Long Short-Term Memory networks are showing significant promise in processing complex spatiotemporal data for accurate climate and environmental predictions [24], a direction that can greatly benefit extreme-value forecasting.

**Perspective takeaway.** Sensor networks are most valuable when tightly coupled to hybrid models and decision logic (alerts, adaptive sampling), ideally embedded in an operational/digital-twin loop for intervention—not only monitoring [22].

## 4. Open Challenges (2025)

**C1. Uncertainty & extremes.** Routine reporting of predictive intervals for maxima remains rare; hybrid pipelines need calibrated UQ (e.g., conformal or Bayesian layers) to be trustworthy in operations.

**C2. Reproducibility & benchmarks.** The community lacks open datasets that pair raw sensor streams, CFD/RANS snippets, geometry metadata, and ground-truth maxima; this hinders fair comparison.

**C3. Sensor layout optimization for extremes.** Optimal placement under budget/latency constraints is unsolved—especially with flow non-stationarity and multi-hazard objectives [23].

**C4. Real-time STE under complex morphologies.** Robustness to turbulence transients and background bias requires STE pipelines that combine physics-informed priors with adaptive surrogates [25].

**C5. Edge deployment & energy budget.** Running inference near sensors is attractive but constrained by power, compute, and model compression; neuromorphic/edge AI is promising yet immature for urban dispersion [19].

**C6. Operational/digital twins beyond monitoring.** Most urban DTs remain prototypes; governance, data standards, and actionable intervention loops are still emerging [22].

## 5. Research Agenda 2025–2030

**R1. Open, task-oriented benchmarks for extremes.** Publish datasets that tie sensor streams + flow surrogates + maxima labels under shared licenses, with leaderboards for maxima prediction and STE. A minimal viable dataset for such a benchmark would include hourly measurements from an urban air quality sensor network over a typical week, paired with short-term (e.g., 24-h) CFD-RANS simulations of the area under different wind directions, along with building geometry and annotated ground-truth maxima for specific receptor locations.

**R2. Physics-informed learners for extremes.** Embed conservation constraints and stability priors in learners; explore extreme-value-aware losses and tail-calibrated objectives [21].

**R3. Adaptive STE.** Couple MCMC/variational back-ends with dynamic-mode surrogates for unsteady flow; support on-the-fly bias correction from mobile sensors [25].

**R4. Edge-ready inference.** Distill hybrid models to run on microcontrollers/neuromorphic chips with duty-cycling; quantify accuracy-vs-energy trade-offs in field pilots [19]. A practical target for a breakthrough accuracy-energy balance would be maintaining the maximum concentration prediction error below 15% compared to a desktop reference model, while consuming less than 100 mW during an inference cycle on a common microcontroller (e.g., ARM Cortex-M).

**R5. Interoperable digital twins.** Standardize I/O for CFD–sensor fusion, including uncertainty channels; integrate with live decision rules (AQ alerts, evacuation, micro-siting) [26].

**R6. Reporting standards.** Adopt minimal checklists (data provenance, geometry, sensor calibration, UQ disclosure) to raise reproducibility across studies of environmental extremes.

## 6. Evaluation and Discussion

The empirical methodology presented in this work has been applied across multiple environmental domains with promising results. In this chapter, we evaluate the performance of the model in terms of its predictive accuracy, generalizability, and computational efficiency. The discussion draws upon a range of case studies and previously published works that span applications in wind energy forecasting, pollutant dispersion modeling, and meteorological extremes.

### 6.1. Accuracy and Comparison with High-Fidelity Models

One of the main strengths of the empirical formulation lies in its ability to estimate peak values using minimal input data. Comparisons with high-resolution simulations, such as CFD-RANS and LES, have demonstrated strong agreement between predicted and simulated maxima. In prior studies, the empirical model captured maximum wind speeds and pollutant concentrations within a relative error margin of less than 10%, while requiring only a fraction of the computational resources.

For example, in applications involving urban pollutant dispersion, the model’s estimates of peak concentrations based on statistical descriptors of the flow field (mean, standard deviation, and correlation time) aligned closely with values obtained through full RANS simulations. Similarly, for wind energy applications, the model successfully reproduced maximum energy output predictions from CFD-derived wind speed distributions across diverse terrains, validating its applicability in both complex and simplified environments.

### 6.2. Statistical Performance Metrics

Quantitative evaluation using standard performance indicators further supports the robustness of the approach. Across various datasets, the model exhibits high Pearson correlation coefficients (R > 0.9) between predicted and actual maxima. Root Mean Square Error (RMSE) and Mean Absolute Error (MAE) remain consistently low, underscoring the model’s ability to generalize across spatial and temporal scales.

In one case involving atmospheric pressure extremes, the empirical predictions achieved RMSE values below 2 hPa when benchmarked against reanalysis data. Similarly, temperature peak estimation during heatwave conditions was successfully implemented using the same framework, with negligible bias.

### 6.3. Model Sensitivity and Parameter Influence

Sensitivity analysis reveals that the model’s output is most strongly influenced by the standard deviation and correlation time of the input time series. This observation is consistent with the physical interpretation of transient fluctuations and their role in shaping extreme values. The mean value sets the baseline, but it is the variability and persistence in time that drive the magnitude of extremes.

Care must be taken, however, in defining the correlation time scale τ, especially when data are noisy or irregular. In such cases, smoothing techniques or adaptive windowing may be necessary to ensure stability and consistency in the estimation.

### 6.4. Advantages, Limitations, and Use Cases

The key advantages of the proposed approach include:

**Computational efficiency**, enabling fast predictions without the need for full simulations

**Scalability**, applicable from local receptor-level exposure to regional energy planning

**Ease of calibration**, as it leverages readily available sensor data or existing CFD outputs

**Transferability**, with successful tests across multiple environmental variables

Nonetheless, the empirical approach has certain limitations. It does not capture detailed flow structures, chemical interactions, or sub-grid phenomena. It also assumes statistical stationarity over the time window of analysis, which may not hold in rapidly changing environments. For this reason, the method is best used as a complement to high-fidelity modeling or as a rapid screening tool for decision support. Research towards **Adaptive Source Term Estimation (R3)**, which integrates dynamic models and real-time data, aims precisely at addressing this limitation by enabling the model to adapt to non-stationary conditions.

**Use Cases in Operational Settings**

The model is particularly suited for several real-world applications:

**Rapid Emergency Response:** Estimating peak pollutant exposure during industrial accidents or hazardous releases.

**Wind Energy Portfolio Management:** Predicting extreme wind power outputs for grid stability and storage planning.

**Urban Planning and Zoning:** Identifying hotspots for high pollution or wind loads to guide architectural and regulatory decisions.

**Mobile Sensor Routing:** Dynamically guiding mobile sensors or drones to regions of predicted extremes for validation or enhanced monitoring.

In each case, the model serves as a computationally efficient surrogate, complementing high-fidelity tools for scenarios requiring speed and adaptability.

### 6.5. Discussion on Broader Impacts and Integration Potential

The integration of this empirical approach into operational tools can significantly enhance real-time forecasting, early warning systems, and environmental risk assessment. Its ability to interface with sensor networks opens new avenues for adaptive environmental control, while its low computational cost makes it ideal for embedded systems or resource-constrained scenarios.

In the broader scientific context, the model contributes to the growing body of hybrid techniques that seek to bridge data-driven and physics-based methodologies. As environmental systems become increasingly monitored and digitized, such empirical models offer a pragmatic path forward—one that balances speed, accuracy, and interpretability.

**Comparison to recent literature.** The proposed hybrid stance aligns with evidence that physics-AI combinations can surpass traditional forecasts for spatiotemporal extremes while reducing computational cost; similarly, recent STE studies emphasize model-based priors plus data-driven acceleration for practical performance in urban morphologies. Emerging digital-twin applications support intervention-oriented workflows, yet reproducibility and governance remain outstanding [21].

## 7. Outlook

Extreme-value prediction is ready for a pragmatic turn: compact, physics-guided learners trained on sensor-CFD pairs; uncertainty-aware reporting; and digital-twin loops that enable interventions, not just monitoring. The six-point agenda outlined here—benchmarks, physics-informed learners, adaptive STE, edge inference, interoperable twins, and reporting standards—offers a concrete path for 2025–2030. Progress hinges on open datasets and cross-domain collaboration between atmospheric science, fluid mechanics, and the sensor-networks community.

Looking ahead, several avenues emerge for future development. First, extending the empirical framework to incorporate spatial correlation metrics may improve its applicability in complex urban topographies. Second, dynamic adaptation of the correlation time scale using machine learning or data assimilation could enhance the robustness of the model in rapidly changing environments. Third, embedding the model into real-time decision-support systems—particularly those powered by distributed sensor networks—can bridge the gap between predictive modeling and environmental management.

Moreover, the empirical methodology lends itself to broader integration within hybrid digital twins for environmental systems, where fast surrogates are necessary for operational control. Its generalizability opens opportunities for application in other time-dependent phenomena, such as solar radiation forecasting, urban heat island analysis, and risk assessment under climate extremes.

In summary, the proposed empirical framework represents a significant step toward fast, reliable, and interpretable prediction of environmental extremes. As environmental data streams become more abundant and systems more interconnected, such lightweight yet effective models can play a central role in advancing resilience, sustainability, and informed decision-making across disciplines.

## Data Availability

Dataset available on request from the authors. The raw data supporting the conclusions of this article will be made available by the author on request.
